# Adolescents’ sleep quality during the COVID-19 pandemic

**DOI:** 10.5935/1984-0063.20220025

**Published:** 2022

**Authors:** Brigitte Bezerra Lima da Silva, Maria Carolina Ferreira de Melo, Luciana Moraes Studart-Pereira

**Affiliations:** Universidade Federal de Pernambuco, Departamento de Fonoaudiologia - Recife - PE - Brazil.

**Keywords:** Sleep deprivation, Adolescents, Students, Pandemic, Coronavirus

## Abstract

**Objective:**

To assess the sleep quality of Brazilian adolescents during the COVID-19 pandemic.

**Methods:**

Observational, analytical, cross-sectional study with data from a questionnaire on individual characteristics and health, Epworth Sleepiness Scale and the Pittsburgh Sleep Quality Index (PSQI). 153 adolescents participated - 61.4% females, 38.6% males, aged 13 to 18 years, enrolled at school.

**Results:**

58.2% worsened their sleep quality during the pandemic. In the PSQI, 22 (14.4%) had a good, 104 (68%), a poor sleep quality, 27 (17.6%), suggestive of sleep disorders. Excessive daytime sleepiness occurred in 44.4% of the students. Significant associations appeared between sleep and decreased school motivation (p = 0.005), and between sleep and sex (p = 0.015). The pandemic affected more the females - 25.5% reported worse sleep quality, 67% had PSQI scores suggestive of sleep disorders; 46 (30.1%) students reported stress, anxiety, sadness -76.1% were girls.

**Discussion:**

Adolescents had impaired sleep during the COVID-19 pandemic, reinforcing their sleep must be assessed in critical periods.

## INTRODUCTION

Initially detected in Wuhan, China, in 2019, the new coronavirus (SARS-CoV-2) quickly reached the status of a pandemic, that impacted the world population’s economy and health in unprecedented proportions.

The economic tension, the fear of the disease caused by the coronavirus, and the uncertainty about the future are making many people experience stress, with symptoms of depression and anxiety, which are intensified by the social distancing and confinement necessary to prevent the contagion. These issues negatively influence sleep quality, affecting people’s daytime resilience and emotional functioning. In this sense, studies have turned their attention to identifying changes in sleep patterns of specific populational groups to evaluate the extent of the problem^1^. The COVID-19 pandemic negatively impacted young people’s sleep quality; hence, interventions to promote family well-being should have been provided since the beginning of the period of isolation^2^.

Amid the adaptations required to control the coronavirus contagion, the main change in the routine of students was the full-time use of online tools to attend classes and do their academic activities. It must be highlighted, though, that digital activity, despite being familiar to this age group, used to be only a facilitating resource in the learning process. A study observed the youth’s behavior in social isolation and verified circumstances such as worsened school achievements, increased aggressiveness, distress complaints, psychological suffering, and sleep pattern changes^3^.

Sleep is a mechanism that regulates and recovers biological and cognitive functions. Its main purpose is to maintain the normal parameters of the nervous system. A poor sleep quality or lack of sleep affects the quality of life in any age group, possibly having a negative impact on cognitive performance, mood, memory, concentration, learning, logical reasoning, and creativity, reducing immunity and increasing the risk of cardiovascular diseases, diabetes, obesity, and psychiatric diseases^4^; ^5^.

Adolescence is a period characterized by significant physical, emotional, behavioral, and social changes. As for biological phenomena, the sleep pattern commonly changes in this development phase, as young people tend to go later to sleep and wake up later as well^6^. Sleep deprivation in adolescence can have consequences not only to health but also to school obligations and social activities^6^. This phase of life is associated with high stress levels and irregular sleep, as it is a period of intense and decisive school and professional experiences – which, along with the abovementioned factors, intensify the students’ risks of developing behavioral and health disorders^11^.

In this context, investigating young students’ sleep quality in the period of social distancing helps verify this historical moment’s impact on their sleep, making it also possible to propose measures to identify and prevent sleep disorders in this population^3^.

This investigation aimed to assess young people’s sleep quality during the COVID-19 pandemic.

## MATERIAL AND METHODS

This is an observational, analytical, cross-sectional study, approved by the Research Ethics Committee and registered under protocol number 4.068.667. Data were collected via digital means. The sample was selected by convenience, comprising 30.53% (153 adolescents) of the universe of students enrolled at the target schools (501 adolescents). They were adolescents of both sexes, aged 13 to 18 years, enrolled in private schools in the Metropolitan Region of Recife, Pernambuco, Brazil. Students who had some difficulty in filling out the data collection questionnaire personally or who filled it out incompletely were excluded from the study.

The inclusion criteria were as follows: being enrolled in high school, between the ninth and twelfth grades, whose school activities were taking place completely or partially in the remote mode, with intellectual independence to personally answer the questions, and having an electronic device that enabled them to send the questionnaire. Incomplete forms were excluded.

The students started filling out the forms in June 2020 – 3 months (109 days) after in-person classes had been canceled, as a temporary measure to avoid coronavirus contaminations. The in-person classes were canceled by Regulation no. 48809 of March 14, 2020, effective throughout the Brazilian state of Pernambuco, where the research was conducted.

The invitation was disseminated on the schools’ websites and official social media, providing the link to access the electronic questionnaire on Google Docs. The volunteers and their parents/guardians had access first to the informed consent form in the first screen of the electronic questionnaire and only continued participating after both had assented to it. Then, the participants answered a questionnaire on identification data, sleep changes, and motivation to carry out academic activities during the pandemic.

The participants answered the Pittsburgh Sleep Quality Index (PSQI), which assesses sleep quality as good, poor, or suggestive of sleep disorder^7^. The PSQI analysis is based on seven components, each of them with a score ranging from zero (0) to three (3) points. The summed components lead to a total score that ranges from 0 to 21 points. A total score higher than 10 is suggestive of sleep disorder; between 5 and 10, it represents poor sleep quality; and equal to or lower than 4, it means good sleep quality. The final values were calculated following the Scoring Instructions for the PSQI.

Lastly, the students filled in the fields related to the Epworth Sleepiness Scale, a self-administered questionnaire that assesses the likelihood of falling asleep in eight situations involving daily activities, some of them known to be rather soporific. The overall score ranged from zero to 24; scores above 10 points suggest a diagnosis of excessive daytime sleepiness^8^.

A descriptive analysis of the data was conducted with the absolute and percentage frequencies of the categorical variables and with the median, mean, standard deviation, 25^th^ and75^th^ percentile, and minimum and maximum values of the numerical variables of the PSQI scores. The association between two categorical variables was assessed with Pearson’s chi-square test or Fisher’s exact test (when the conditions to use the chi-square were not verified).

We used the Kruskal-Wallis test to assess the difference between the sleep quality categories in relation to the numerical variable (Epworth Scale score), with multiple comparisons of the said Spearman correlation test and Student’s t-test specifically for the null correlation hypothesis. We chose the Kruskal-Wallis and Spearman correlation tests due to the absence of normality of the data. We used the Shapiro-Wilk test for verification.

The 5% margin of error was used for decisions in the statistical tests. The data were entered into Excel spreadsheets and the IMB SPSS, version 23.0, was used for the statistical calculations.

## RESULTS

A total of 153 adolescents participated in the study – 61.4% were females and 38.6%, males, whose ages ranged from 13 to 18 years. The most frequent age was 14 years (30.1%), followed by 13 years (2.0%) and 18 years (9.2%). The other ages ranged from 18.3% to 21.6%.

In the self-assessment, the highest percentage (44.4%) considered their sleep quality fair, the lowest percentages considered it great (5.2%) or horrible (11.1%), and those who considered it poor or good were respectively 19.0% and 20.3% (Table 1). More than half (58.2%) answered that their sleep during the pandemic had grown worse, 31.4% said it had not changed, and 10.4% said it had improved.

**Table 1.** Assessment of the Pittsburgh Sleep Quality Index according to each students’ profile.

Pittsburgh Sleep Quality Index									
Variable	Good		Poor		Suggestive of disorder		TOTAL		p-value
	N	%	N	%	n	%	N	%	
**Age group**									p(1) = 0.261
13 to 15	13	16.9	54	70.1	10	13.0	77	100.0	
16 to 18	9	11.8	50	65.8	17	22.4	76	100.0	
**Sex**									p(1) < 0.001*
Males	17	28.8	39	66.1	3	5.1	59	100.0	
Females	5	5.3	65	69.1	24	25.5	94	100.0	
**High school grade**									p(1) = 0.278
9th grade	10	21.7	29	63.0	7	15.2	46	100.0	
10th grade	4	10.3	31	79.5	4	10.3	39	100.0	
11th grade	5	13.5	25	67.6	7	18.9	37	100.0	
12th grade	3	9.7	19	61.3	9	29.0	31	100.0	
**Considers to be a more active person:**									p(1) < 0.001*
In the morning	7	25.9	18	66.7	2	7.4	27	100.0	
In the afternoon	14	25.9	34	63.0	6	11.1	54	100.0	
At night	1	1.4	52	72.2	19	26.4	72	100.0	
**Considers their sleep quality:**									p(1) < 0.001*
Horrible/Poor	-	-	25	54.3	21	45.7	46	100.0	
Fair	7	10.3	57	83.8	4	5.9	68	100.0	
Good/Great	15	38.5	22	56.4	2	5.1	39	100.0	
**Uses the cell phone before sleeping**									p(1) = 0.245
Yes	20	14.0	96	67.1	27	18.9	143	100.0	
No	2	20.0	8	80.0	-	-	10	100.0	
**Has a chronic disease**									p(1) = 0.107
Yes	1	4.5	14	63.6	7	31.8	22	100.0	
No	21	16.0	90	68.7	20	15.3	131	100.0	
**Has respiratory difficulties**									p(2) = 0.003*
Yes	1	2.3	29	67.4	13	30.2	43	100.0	
No	21	19.1	75	68.2	14	12.7	110	100.0	
**Tiredness during classes**									p(1)<0.001*
Never/rarely	3	30.0	7	70.0	-	-	10	100.0	
Sometimes	14	25.5	40	72.7	1	1.8	55	100.0	
Many times/always	5	5.7	57	64.8	26	29.5	88	100.0	
**Naps during classes**									p(1)<0.001*
Never/rarely	18	20.9	61	70.9	7	8.1	86	100.0	
Sometimes	3	6.7	33	73.3	9	20.0	45	100.0	
Many times/always	1	4.5	10	45.5	11	50.0	22	100.0	
Group Total	**22**	**14.4**	**104**	**68.0**	**27**	**17.6**	**153**	**100.0**	

(*) Significant association at 5.0%; (1) With Pearson’s chi-square test.

The adolescents’ sleep quality, measured with the Pittsburgh sleep quality index, was impaired in most (85.6%) of the group we studied, which explains their daytime tiredness and sleepiness that hindered their activities. There was a significant association (p = 0.005) between self-perceived sleep quality and the data on sleep quality obtained with the PSQI (Table 1).

The daytime sleepiness measured with the Epworth scale was high (>10 points) in almost half the adolescents (44.4%) and had a positive correlation (Spearman correlation = 0.319) with sleep quality, based on the Pittsburgh sleep quality index.

They also reported the possibility of napping in specific situations, with an impact on academic activities. Table 1 shows an association between sleep quality index and tiredness (p<0.001) or the possibility of napping (p<0.001) during class.

Table 2 shows a significant association between the motivation to study and the self-assessment of sleep during the pandemic. There was a higher motivation to study during the pandemic (25.0%) among those who said their sleep had improved. This percentage was 6.3% and 10.1% in the other two sleep assessment categories. The percentage of those who said they were less motivated was higher among those who reported a worsened sleep (67.4%), whereas it was 43.8% and 50.0% in the other two categories. And of those who said their sleep had not changed, 50.0% assessed their motivation as unchanged as well; in the other two categories, it was 25.0% and 25.5%.

**Table 2.** Assessment of sleep changes and motivation to study during the pandemic.

Motivation to study during the pandemic									
Sleep during the pandemic	Improved		Worsened		Unchanged		TOTAL		p-value
	n	%	n	%	n	%	n	%	
Improved	4	25.0	8	50.0	4	25.0	16	100.0	p(1) = 0.005*
Worsened	9	10.1	60	67.4	20	22.5	89	100.0	
Unchanged	3	6.3	21	43.8	24	50.0	48	100.0	
Group Total	16	10.5	89	58.2	48	31.4	153	100.0	

The difference in sleep quality between the sexes was significant –better sleep quality was observed among male participants (28.8%) than female ones (5.3%). It is also observed that females had a higher suggestive percentage of sleep disorder (25.5%) than males (5.1%). When poor sleep is summed with results suggestive of sleep disorders, the percentage increases to 71.2% in males and 94.7% in female young people. Thus, it was analyzed that the girls underwent more significant changes than the boys (Table 1).

Table 3 shows a significant percentage of girls that reported their sleep had grown worse during the pandemic. The percentage of those who answered their sleep had grown worse during the pandemic was higher among females (67.0% x 44.1%) and the percentage of those who assessed their sleep as unchanged was higher among males (44.1% x 23.4%). It was further identified that 46 (30.1%) of the 153 participants of the study spontaneously reported symptoms of stress, anxiety, and sadness – of whom 23.9% were boys and 76.1%, girls.

**Table 3.** Assessment of sleep changes during the pandemic in relation to sex.

	Sex						
The sleep during the pandemic	Males		Females		Group Total		p-value
	n	%	n	%	n	%	
Improved							p(1) = 0.015*
	7	11.9	9	9.6	16	10.5	
Worsened	26	44.1	63	67.0	89	58.2	
Unchanged	26	44.1	22	23.4	48	31.4	
**TOTAL**	**59**	**100.0**	**94**	**100.0**	**153**	**100.0**	

(*) Significant association at 5.0%; (1) With Pearson’s chi-square test.

Sleep quality was also associated with the part of the day when the participants feel more active to perform their activities. Almost half of those researched (47.1%) said they are more active at night, followed by 35.3% that said they are so in the afternoon, and only 17.6%, in the morning. Of those who are more active at night, 26.4%had a suspicion of sleep disorder, 72.2% had poor sleep according to the PSQI, and only1.4% had a good sleep quality. When asked about habits that affect sleep, a little more than half of them (53.6%) admitted having routines such as practicing extenuating exercises, heavy meals soon before going to bed, using electronic devices, taking medications, having energy drinks – all of which affect sleep. The vast majority (93.5%) answered they used their cell phones before sleeping, without association with sleep quality.

Concerning health self-assessment, 45.8% of this study’s population considered it good, 24.2% considered it fair, and 22.9% reported great health. The percentage of those who reported having a chronic disease was 14.4%, while 28.1% said they had respiratory difficulties. Table 1 reveals a significant association (p = 0.003) between respiratory difficulties and poor sleep quality and suspicion for a sleep disorder. The percentage with good sleep quality was higher among those who did not have respiratory difficulties than those who did (19.1% x 2.3%), whereas the percentage of those with a sleep disorder was higher among who reported respiratory difficulties than who did not (30.2% x 12.7%).

The PSQI component related to sleep duration was analyzed separately and revealed that only 33.3% of the participants slept more than 7 hours a day, which is the PSQI cutoff score. However, the scores of this protocol refer to adults.

## DISCUSSION

Sleep quality is influenced by social factors (such as the type of occupation and housing), biological factors (such as age, sex, chronotype, and health problems), behavioral factors (such as eating habits, physical activities, smoking and drinking habits, and sleep-related routine – which involves academic demands and use of electronic devices before going to bed)^3^. In the context of the pandemic, the emotional factors also stand out, as they are related to uncertainties and social distancing^4^.

In this investigation, influenced by biological or behavioral factors, the self-perception of sleep was coherent with the PSQI assessment. The adolescents that considered their sleep quality poor or horrible had a PSQI that corresponded to poor sleep quality or suggestive of sleep disorder. Likewise, 38.5% of those who considered their sleep good or great had their self-assessment confirmed by the sleep quality screening.

Most of the participants (58.2%) reported their sleep changed to worse during the pandemic – similar to worldwide research^9^ with respondents from 49 countries, in which 48% perceived a decrease in sleep quality in comparison with before the coronavirus pandemic. We assessed them with the PSQI and verified that 85.6% had poor sleep quality, and 44.4% had excessive daytime sleepiness, based on the Epworth scale. Most students are known to prefer nighttime activities, which may cause daytime sleepiness and have consequences on their quality of life^10^. In the present paper, we observed these effects in the frequent naps and tiredness during classes. Moreover, excessive daytime sleepiness may be associated with motivation to study, high psychological demands due to this period, and the Brazilian schools’ inadequate morning schedule, which help understand that daytime sleepiness and sleep quality are multifactorially influenced^3.^

The results indicated a relationship between less motivation to study and worsened sleep during the pandemic, highlighting the interference of sleep quality on physical and mental disposition. Sleep deprivation in children and adolescents causes frustration, sleepy/ uninterested behavior, low tolerance for conflicts, inattention, and difficulties following routines or dismissing themaltogether^6^. A study carried out with university students in Brazil during the COVID-19 pandemic found those young people were discouraged to perform their academic and everyday activities, besides their complaints related to missing the school setting and in-person interaction with their classmates because of social distancing^11^.

The females had higher indexes suggestive of sleep disorders (25.5%), higher self-perception of sleep quality, as well as a higher prevalence of worsened sleep during the pandemic (67%). On the other hand, a considerable percentage of the boys (44.1%) reported not noticing any changes. Usually, self-assessing and reporting health problems are more frequent among females than males; therefore, they use health services more often^12^. Also, there was a higher percentage of girls who spontaneously reported signs of anxiety, stress, and sadness. This confirms information of a recent study^13^that points to this population (along with young adults and people with a history of depression) as one of the most affected by these conditions during the pandemic. The said paper also indicates that females have a higher prevalence of increased overall anxiety due to COVID-19 than males.

The sleep-wake cycle respects patterns that vary according to the person’s age, sex, and biopsychosocial characteristics. The chronotype refers to each person’s inner regulation in terms of preferred time to do their tasks and daytime activities, as well as go to bed and wake up^6^. The phase delay is a mechanism characterized by the need to go to sleep later and consequently wake up later. This phase commonly takes place in adolescence due to biological, behavioral, social, or academic changes. Therefore, adolescence is a greatly vulnerable age to develop sleep disorders, such as insomnia^14^. Social isolation as a COVID-19 preventive measure has worsened the circadian misalignment, as the changes in routine affect the effectiveness of sleep and its capacity to regulate the metabolism, with consequences to the human body, making it more vulnerable to infections, including that of SARS-CoV-2^9^.

This study showed that approximately 50% of the participating adolescents preferred to carry out their activities at night. Adolescents with Circadian Rhythm Sleep Disorders (CRSD) are at greater risk of having symptoms of depression and anxiety, in addition to cognitive difficulties, which affect their school achievement^14^. The preventive measures and changes in routine affect more intensely the adolescents who already tend to a delayed sleep-wake cycle. However, this aspect will not be a noticeable problem to the students while their routines remain this way. In this context, it is important to raise awareness about the relationship between the adequate sleep-wake cycle, the immune system, and the maintenance of overall health quality^15^.

The need to adapt the routine of activities to one’s biological rhythm is a broadly discussed topic. The Brazilian Sleep Society verified in 2019 that the timetable in most of the Brazilian schools is inadequate to the young people’s sleep needs, as the activities begin before eight o’clock in the morning. Such a schedule can cause sleep deprivation and consequently impair their biological and emotional regulation and academic performance^11^.In this research, 86.7% of the participants who had obligatory academic activities in the morning either reported poor sleep quality or had a PSQI result suggestive of sleep disorder.

The habit of excessively using the smartphone is related to a worse sleep quality^16^. Using the cell phone before going to sleep was analyzed separately in this study, as it is a habit that affects sleep and is rather common in the adolescents’ routine. It was verified that everyone who had a poor sleep quality or was suggestive of sleep disorder used the cell phone before sleeping. The social and academic demands during the pandemic justify the increased connectivity and intense use of digital tools. However, the additional screen time with these devices is directly associated with changes in sleep quality, time to go to sleep, and sleep pattern regularity^17^.

Besides the abovementioned behavioral issues, 30.2% of those assessed as suggestive of sleep disorder complained of respiratory difficulties and/or allergic rhinitis or asthma. According to a study^18^, patients with these types of respiratory conditions have higher scores of sleep disorders, sleep latency, and impaired sleep quality. Also, analyzing the PSQI results, the research indicated that people with allergic rhinitis are likewise more likely to having insomnia, enuresis, restless sleep, obstructive sleep apnea, and snoring.

Analyzing separately the PSQI component related to sleep duration, 66.7% of the participants were identified as sleeping less than 7 hours a day (mean of 6 hours and 43 minutes), which is considered inadequate based on the instrument’s cutoff score. Considering that they are adolescents, these are even more alarming data, as this population should sleep a mean of 8 to 10 hours a day^19^. A study^20^conducted with Brazilian university students also identified a mean of 6.5 hours of sleep on weekdays. Sleep deprivation is a recurring factor in epidemic events that require social isolation. Therefore, maintaining habits (such as healthy eating habits, physical activities, and sleep hygiene) is essential to minimize negative effects on health during this time^21^; ^22^.

The study is limited because it was carried out with adolescents only from private schools and it did not stratify the participants’ socioeconomic level. In any case, the issues reported here furnished an overview of the record and assessment of young people’s sleep quality at times of social isolation. The results are essential to formulate attention, treatment, and prevention strategies in similar circumstances.

This study verified a sharply impaired sleep quality in adolescents during the period of social isolation due to the SARS-CoV-2 pandemic in 2020. The main aspects identified were worsened sleep quality, increased use of digital tools, more sleep-related complaints among females in the period, less motivation to study, and hours of sleep below the recommended for this age group in question.

Considering the involvement of physiological, psychosocial, and environmental aspects related to sleep disorders, the importance of assessing these young people’s sleep conditions in critical periods such as the one experienced in the SARS-CoV-2 pandemic is reinforced.
